# Progress and future directions of biogeographical comparisons of plant–fungal interactions in invasion contexts

**DOI:** 10.1111/nph.70228

**Published:** 2025-05-21

**Authors:** Arpad E. Thoma, Ylva Lekberg, Dávid U. Nagy, Min Sheng, Erik Welk, Christoph Rosche

**Affiliations:** ^1^ Institute Biology, Department of Geobotany and Botanical Garden Martin Luther University Halle‐Wittenberg 06108 Halle Germany; ^2^ MPG Ranch Missoula MT 59801 USA; ^3^ Department of Ecosystem and Conservation Sciences, W.A. Franke College of Forestry and Conservation University of Montana Missoula MT 59812 USA; ^4^ Plant Evolutionary Ecology, Institute of Ecology, Evolution and Diversity, Faculty of Biological Sciences Goethe University Frankfurt 60438 Frankfurt am Main Germany; ^5^ College of Forestry Northwest A&F University Yangling Shaanxi 712100 China; ^6^ German Centre for Integrative Biodiversity Research (iDiv) Halle‐Jena‐Leipzig 04103 Leipzig Germany

**Keywords:** ecological sampling design, environmental heterogeneity, native vs nonnative range comparisons, plant invasion, plant–fungal interactions, sampling bias, sampling quality, spatio‐environmental variation

## Abstract

Plant invasions are biogeographical phenomena that may involve shifts in belowground plant–fungal interactions, such as the release from fungal pathogens or more beneficial interactions with mutualists in nonnative ranges. However, native and nonnative ranges are not uniform but environmentally heterogeneous, and plant–fungal interactions are strongly shaped by spatio‐environmental context. Intense discussion at the 45^th^ *New Phytologist* Symposium revealed that we lack information on how well spatio‐environmental variation *within* ranges has been considered in samplings and analyses of studies comparing plant–fungal interactions *between* ranges. Through a systematic review, we assessed the sampling quality of recent biogeographical studies. We found that the majority relied on a limited population sampling within each range, often covering only a small fraction of the species' spatial distribution and macroclimatic niche. Additionally, low similarity between the sampled climatic gradients in the native and nonnative ranges might have introduced false‐positive differences across ranges. These sampling deficiencies may undermine the robustness and representativeness of range comparisons, thereby restricting our ability to accurately assess the role of plant–fungal interactions in invasion success. We recommend that future research incorporate broader and more comparable spatio‐environmental variation in both ranges, and we provide practical guidelines for improving sampling designs.

## Introduction

Plant invasions are fundamentally biogeographical phenomena that occur when a species is introduced from its native range, where populations are regulated by specific environmental factors, to a nonnative range, where novel factors shape its abundance (Hierro *et al*., 2005). Many successful nonnative plants thrive due to altered biotic interactions in their introduced ranges, often leading to greater performance of nonnative than native populations (reviewed by Parker *et al*., 2013). In this context, belowground shifts in plant–fungal interactions have frequently been identified as key drivers of invasion success (Mitchell *et al*., 2006; Pringle *et al*., 2009; Inderjit & van der Putten, 2010). These shifts may be driven by range distributions of fungal pathogens and mutualists (Tedersoo *et al*., 2014; Pearson *et al*., 2022; Sheng *et al*., 2022), altered function of fungi (Wolfe & Pringle, 2012), or modifications in plant traits, such as root exudates that influence fungal associations (Inderjit *et al*., 2021; Tian *et al*., 2021; Yu *et al*., 2022). Comparative biogeographical studies have greatly advanced our understanding of these processes and highlighted the role of soil fungi in promoting plant invasions (Reinhart & Callaway, 2006). However, current conceptual frameworks emphasise that experimental studies in ecology require broad sampling across diverse environments (Schweiger *et al*., 2016). This also applies to plant invasions and specifically to comparisons across native and nonnative ranges (Colautti & Lau, 2015; Lucas *et al*., 2024), yet we have no information on the sampling quality of recent biogeographical studies on plant–fungal interactions.

Over the decades, various hypotheses have been proposed to explain plant invasion success through altered plant–fungal interactions. Key examples include the escape from specialist pathogens (Dawson & Schrama, 2016) and changes in interactions with generalist pathogens that may spill over to reduce the competitiveness of the native neighbours (Flory & Clay, 2013; Waller *et al*., 2024). In addition, invasive plants can make better use of mutualists in their nonnative than in their native range (Reinhart & Callaway, 2006) by associating with more or different fungi (Sheng *et al*., 2022; Yu *et al*., 2022) or disrupt existing local mutualistic interactions of resident native plants (Stinson *et al*., 2006). Furthermore, plants can adapt to the novel biotic interactions over very short evolutionary scales (van Kleunen *et al*., 2018). Such rapid evolution can lead to differentiation between native and nonnative populations, often resulting in greater performance of the nonnative populations (Bossdorf *et al*., 2005). However, biotic interactions and plant performance vary within ranges due to environmental conditions as well as spatial and temporal co‐occurrence patterns between plants and fungi. As such, biotic interactions and plant performances often display patterns that are neither uniform nor static (Kožić *et al*., 2024; Nagy *et al*., 2024; Rosche *et al*., 2025). This could lead to incorrect conclusions of range differences, especially in cases where sampling designs do not consider the broad spatio‐environmental context of study species (Colautti & Lau, 2015).

To investigate biogeographical differences in plant–fungal interactions, researchers often survey naturally occurring populations of the target invasive plant and record its performance (e.g. biomass of shoots or roots or number and size of seeds) and fungi in either its roots or rhizosphere soil using microscopy or molecular techniques. Experimental approaches may involve field applications of fungicides or the use of soil and seeds for subsequent greenhouse or growth chamber studies. These latter types of studies often use a plant–soil feedback approach where soil biota are amplified by native and invasive plants and then used as inoculum for a second round to assess effects on plant performance (van der Putten *et al*., 2013). Field experiments typically require simultaneous set‐ups in both ranges to avoid introducing novel genotypes, whereas greenhouse or growth chamber experiments can be conducted with caution in a single range. When studying plant–fungal interactions, careful sampling across ranges is particularly critical for several reasons. First, fungal species diversity can increase linearly with area sampled, especially in environmentally heterogeneous regions (Peay *et al*., 2007). Consequently, if native and nonnative ranges are sampled at different spatial scales, observed differences in fungal community dispersions could reflect sampling bias rather than true biogeographical patterns. Second, global surveys have shown that fungal communities are highly sensitive to abiotic gradients such as climate, soil pH, and nutrient availability (Tedersoo *et al*., 2014; Davison *et al*., 2015; Lekberg *et al*., 2021). To avoid region‐ or habitat‐specific (i.e. unrepresentative) results, it is thus essential to sample across large extents of the environmental niches of the study species (reviewed by Lucas *et al*., 2024). Third, the outcomes of plant–fungal interactions are inherently context‐dependent (Bennett & Groten, 2022; Singh *et al*., 2023). As such, observed differences in fungal community composition and their effect on plant performance between native and nonnative ranges may not necessarily indicate altered biotic interactions but could instead result from differences in environmental contexts sampled in either range.

Together, these three pitfalls illustrate how inadequate sampling can lead to unrepresentative results, including false‐positive differences between native and nonnative ranges. This viewpoint aims to assess the current state of biogeographical studies on plant–fungal interactions and to identify methodological limitations the existing research might have. Specifically, we reviewed relevant publications to (1) report characteristics of comparable studies on plant–fungal interactions (e.g. target plant species, population number, environmental conditions measured at sampling sites) and (2) evaluate how well the samplings captured three key aspects: (2.1) the spatial coverage within ranges; (2.2) the climatic coverage of niches of the focal plants within ranges; and (2.3) the climatic similarity of niches sampled between the ranges. To address these aspects, we asked the following questions: are samplings more comprehensive in native or nonnative ranges? Are single‐species studies conducted with better sampling designs than multi‐species studies? Has sampling quality improved over time? Based on our findings, we (3) outline approaches we believe could improve the study design of native vs nonnative range comparisons.

## Current state of comparative research on plant–fungal interactions

**Fig. 1:**
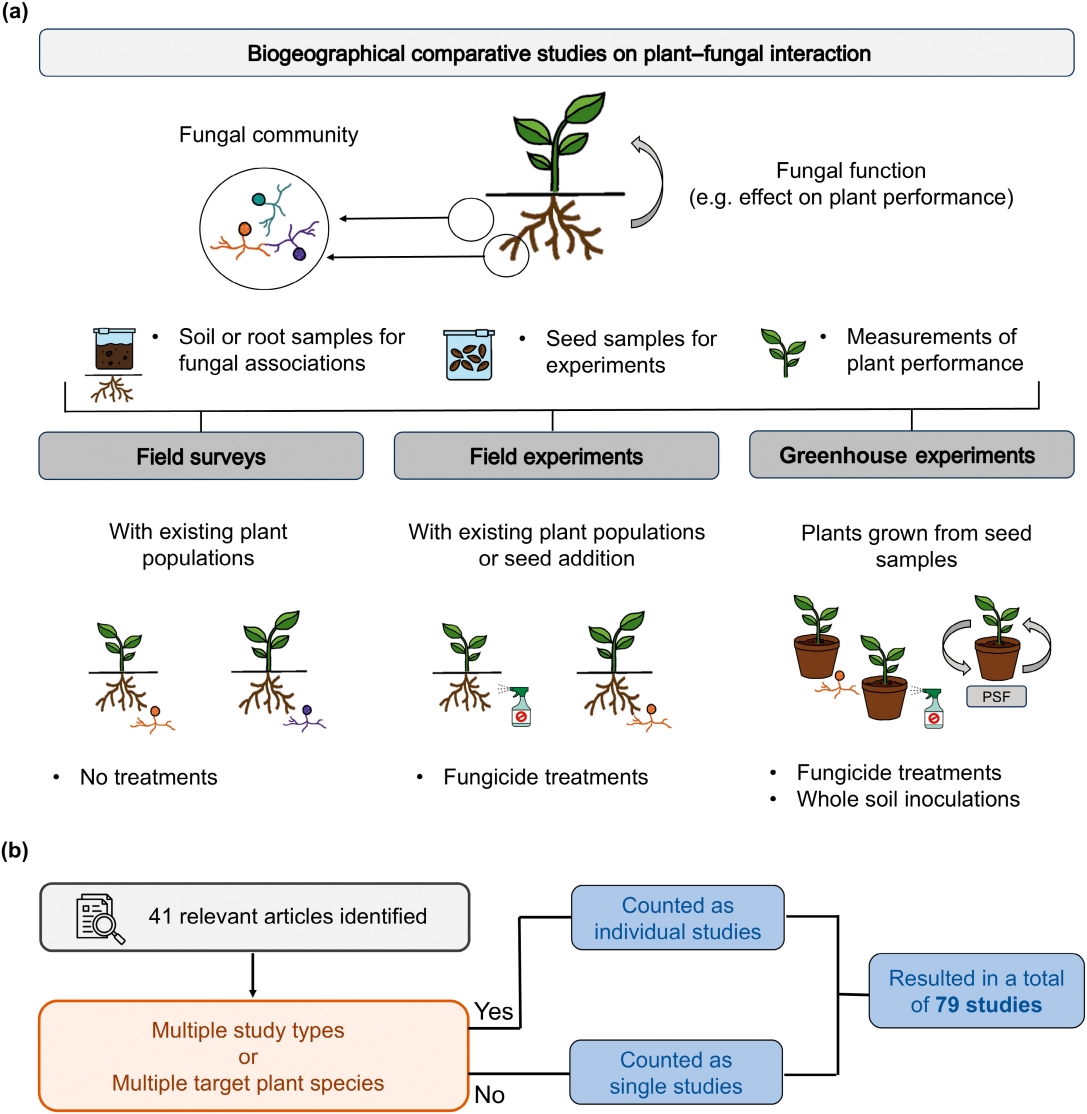
Conceptual overview of the selection of articles and studies included in our review. (a) Our review targeted articles that compared fungal community composition or their function (e.g. their effect on plant performance) across the ranges of the study plant species. Common features among these studies included the collection of soil or root samples to assess fungal associations, measurements of plant performance to evaluate fungal effects, or the collection of seeds for use in experiments. The studies varied in design and approach, encompassing three different study types: field surveys, field experiments, and greenhouse experiments. (b) In total, we identified 41 relevant articles, including 79 individual studies. If articles involved multiple study types or multiple target plant species, they were counted as individual studies. Otherwise, they were counted as single studies. Further details on the process of the systematic literature review and the entire literature screening are provided in Supporting Information Notes S1, Figs S1 and S2.

We conducted a systematic literature review of biogeographical studies published since 2000 that compared plant–fungal interactions between the native and nonnative ranges of invasive plant species. Our review targeted studies that compared fungal community composition or their function (i.e. their effect on plant performance) across the ranges of the study plant species (Fig. 1a). Included studies examined plant performance in relation to soil fungi, either through fungicide treatments or whole‐soil inoculations from the native or nonnative range (for detailed information on our literature search, see Supporting Information Notes S1; Fig. S1). We included studies using whole‐soil because fungi arguably constitute one of the most important groups of soil‐borne pathogens (Raaijmakers *et al*., 2009) and mutualists (Smith & Read, 2010; Delavaux *et al*., 2017).

Our literature search identified 1625 articles, of which most were excluded after title and abstract screening, leaving 172 articles for further evaluation. The full‐text screening excluded 131 publications because they were reviews, clearly addressed a different topic, or lacked range comparisons within their experiments or surveys (see Fig. S2 for details on our screening). In total, we identified 41 relevant articles. Some of the articles conducted multiple study types (e.g. field surveys, field, or greenhouse experiments) or studied multiple plant species. In these cases, we considered multiple individual studies within an article, resulting in a total of 79 studies (Fig. 1b; Table 1). To account for the potential dependency of studies within the same paper, we included paper as a random effect in our linear mixed‐effects models.

**Table 1:** Summary of information extracted in our literature survey.

Extracted information	Characteristics	No. of studies	(%)
No. of plant species investigated	Single species	37	46.8
	Multiple species	42	53.2
Study type	Field survey	31	39.3
	Field experiment	8	10.1
	Greenhouse experiment	40	50.6
Soil origin for greenhouse experiments	Single location	9	22.5
	Multiple locations	31	77.5
Soil from multiple locations	Pooled	7	22.6
	Not pooled	24	77.4
Abiotic factors measured at sampling site (*in situ*)	Yes	47	59.5
	No	32	40.5
Environmental conditions discussed to explain results	Yes	34	43
	No	45	57
Primary objective in soil biota	Surveyed fungal communities	35	44.3
	Assessed fungal function	37	46.8
Main research focus	Plant performance	49	62
	Fungi composition or function	72	91.1
Found range difference in plant performance	Yes	42	85.7
	No	7	14.3
Range differences found in fungal composition	Yes	28	80
	No	7	20
Range differences found in fungal function	Yes	28	75.7
	No	9	24.3

The table presents the number of studies and their relative proportions (%) with respect to certain study characteristics. Note that some studies assessed both plant performance and fungi composition or function, and thus the relative proportions in the attribute ‘main research focus’ can exceed 100%. A comprehensive list of reviewed articles and extracted data is provided in Supporting Information Table S1.

Studies either assessed plant performance and root or soil fungal associations within existing populations or collected seeds and soil for controlled experiments (Fig. 1a). As a result, sampling was guided by the plant's distribution rather than that of individual fungal taxa. This approach is reasonable, because even though hypotheses involving shifts in plant–fungal interactions consider fungal distributions (e.g. enemy release), the primary focus remains on the plant invasion. Also, assessing the extent to which niches were sampled requires knowledge of the distribution of individual taxa, which is still largely unknown for fungi despite current efforts to address this issue (e.g. Větrovský *et al*., 2023). A comprehensive list of the reviewed articles and extracted data is provided in Table S1.

In total, 54 invasive plant species were investigated, with some species, such as *Conyza canadensis*, *Triadica sebifera*, and *Centaurea stoebe*, being the focal species across multiple studies (Table S2). Most studies (50.6%) examined soil biota impact on native and nonnative plant performance through soil inoculations from native and/or nonnative ranges or fungicide treatments in controlled greenhouse experiments (Table 1). Of these 40 greenhouse studies, nine used soil from a single location to grow native and nonnative plants. Seven of the other 31 greenhouse studies pooled soils from multiple sites, whereas the remaining 24 studied sampled soil from multiple locations. Field surveys of fungal communities accounted for 39.3% of all studies, whereas field experiments were notably underrepresented (10.1%). This underrepresentation likely relates to logistical challenges and their time‐demanding nature, often requiring extended periods to fully capture ecological interactions and impacts (Dickie *et al*., 2017). Of the 35 studies that surveyed fungal communities, 25 studies assessed fungi in roots and 10 studies assessed fungi in soil.

In their biogeographical comparisons, 70.9% of the studies reported significant differences between native and nonnative populations, either in how fungi affected plant performance or in the composition of associated fungal communities. However, in 74.1% of the studies, these findings were based on fewer than 10 sampled plant populations per range (mean = 6.48, SD = 4.01, Table S2; Fig. S3). Also, despite the critical role of environmental conditions in shaping plant performance, fungal community composition, and plant–fungal interactions (Tedersoo *et al*., 2014; van der Putten *et al*., 2016), only 59.5% of the studies measured abiotic factors, such as soil properties and climate. Even fewer studies (26.6%) incorporated these factors into their analyses. Both the limited number of sampled populations and the lack of environmental data in statistical models may restrict the representativeness of native vs nonnative range comparisons (Rosche *et al*., 2018). At a minimum, the potentially confounding effects of within‐range variation should be considered in discussions of biogeographical comparisons (Lucas *et al*., 2024). However, only a minority of the reviewed studies accounted for this.

## Sampling efforts in biogeographical studies on plant–fungal interactions

**Fig. 2:**
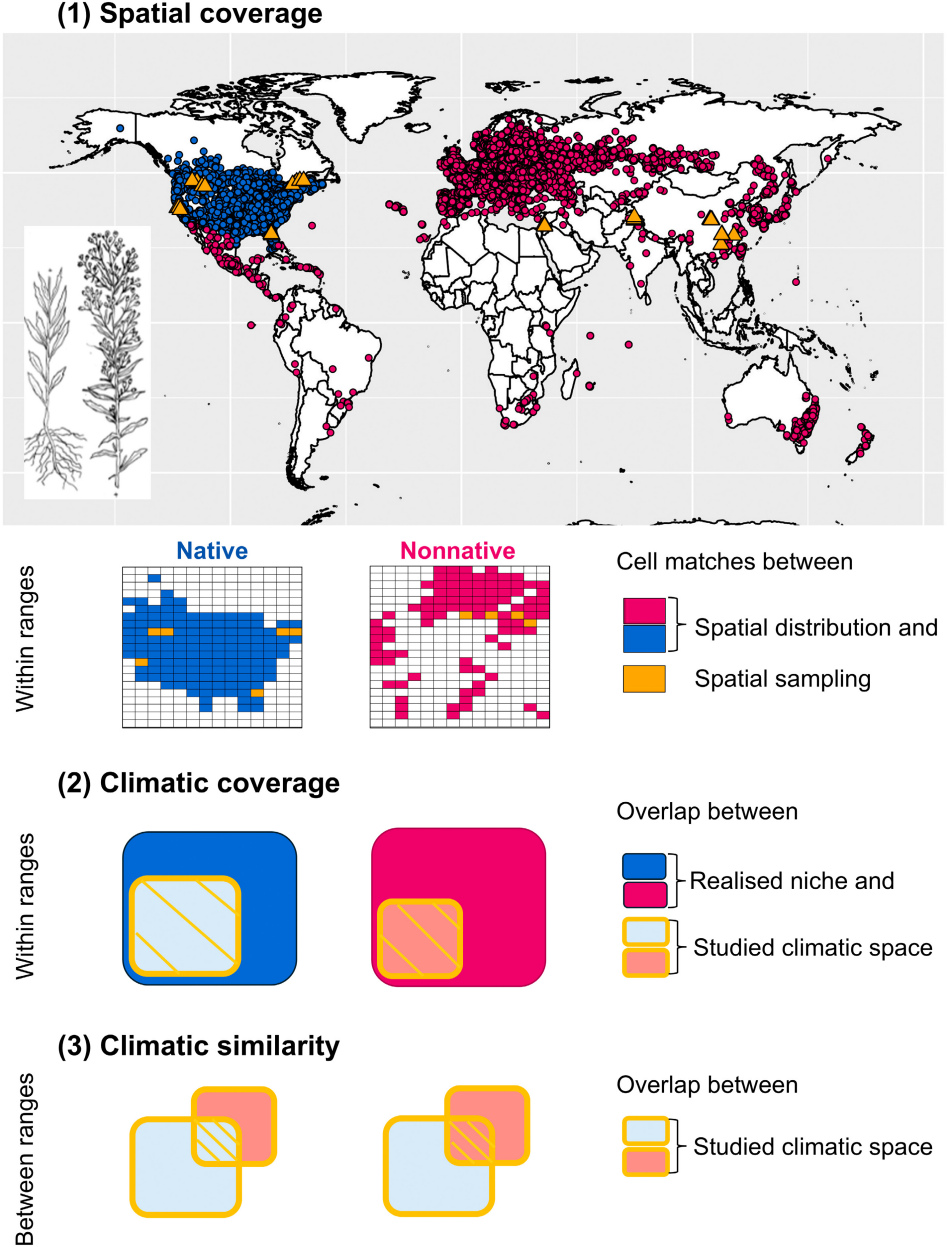
Schematic overview of the calculation of sampling quality parameters. The example illustrates a field survey by Sheng *et al*. (2022) on fungal associations with *Conyza canadensis* populations. The map displays Global Biodiversity Information Facility (GBIF) occurrence data for *C. canadensis* in its native (blue dots) and nonnative (magenta dots) ranges. Range classification of the GBIF occurrences followed the Invasive Species Compendium (ISC; Diaz‐Soltero & Scott, 2014). Sampling locations from Sheng *et al*. (2022) are shown as orange triangles in both ranges. Three parameters estimate the sampling quality. (1) Spatial coverage: GPS coordinates of GBIF occurrences and sampling locations were used to calculate dynamic match coefficients (DMC; Sporbert *et al*., 2019). Dynamic match coefficients represent a measure of cell matches between the sampling locations and the species' global distribution. (2) Climatic coverage: Bioclim variables were extracted to describe the sampled climatic space (sampling locations) and the realised climatic niche (GBIF occurrences). Using dynamic range boxes (DRB; Junker *et al*., 2016), we calculated the overlap between the sampled climatic spaces (orange squares; light blue, native; light magenta, nonnative) and the overall realised niches (larger squares; blue, native; magenta, nonnative). The left overlap (light blue) indicates the proportion of the overall native climatic space covered by the native‐range sampling, while the right overlap (light magenta) represents the proportion of the nonnative climatic space covered by the nonnative‐range sampling. (3) Climatic similarity: DRBs were used to calculate the climatic overlap between samplings in both ranges. The left overlap (light blue) indicates how much of the nonnative climatic space was covered by the native‐range sampling, whereas the right overlap (light magenta) indicates how much of the native climatic space was covered by the nonnative‐range sampling. Further details on the calculation of sampling quality parameters, including a discussion on the limitations of both the GBIF and ISC databases, are provided in Supporting Information Notes S2 and Fig. S4.

A subset of 63 studies provided sampling coordinates, allowing us to quantitatively assess three sampling quality parameters: (1) spatial coverage; (2) climatic coverage; and (3) the climatic similarity of sampled environmental gradients. Spatial and climatic coverage were calculated separately within the native and nonnative ranges, that is, relative to the overall distribution of the target plant species, whereas similarity was assessed between the ranges (Fig. 2).

### Coverage of the spatial sampling

To assess the spatial sampling coverage in both the native and nonnative ranges, we calculated dynamic match coefficients (DMC). The DMC quantifies the overlap between sampling locations and the species' global distribution across different raster resolutions, thereby assessing qualitative and quantitative sampling coverage simultaneously (Sporbert *et al*., 2019). The global distribution was defined using occurrence records from the Global Biodiversity Information Facility (GBIF; datasets are in Table S3). Dynamic match coefficient values range from 0 to 100%, with values close to 100% indicating a nearly complete match between sampling locations and the species' distribution (for details on the calculation, see Figs 2.1, S4; Notes S2).

**Fig. 3:**
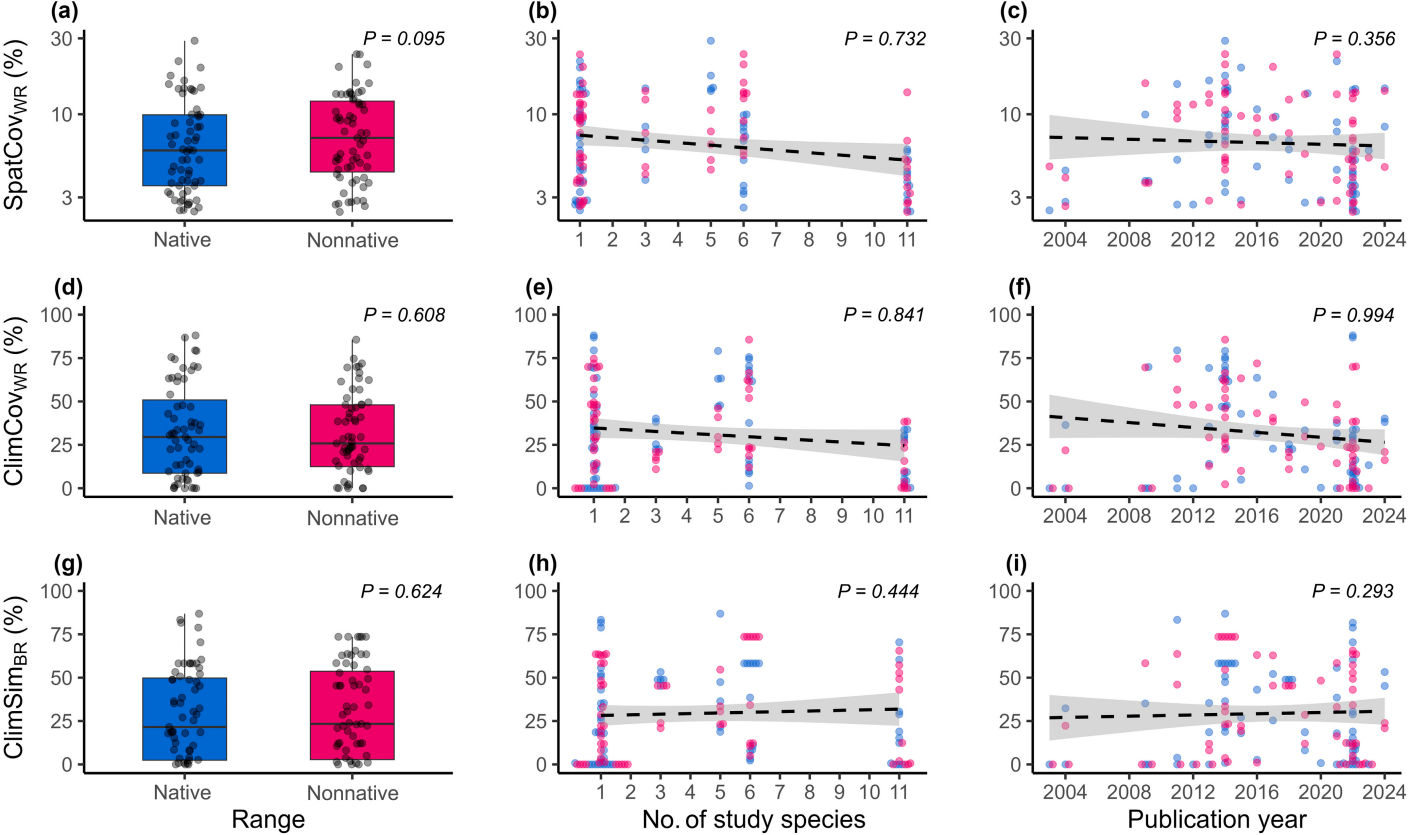
Sampling quality parameters in relation to the range, the number of species studied, and the year of its publication. Relationships are shown for (a–c) spatial coverage within ranges (SpatCov_WR_), (d–f) climatic coverage within ranges (ClimCov_WR_), and (g–i) climatic similarity between ranges (ClimSim_BR_). The climatic similarity boxplots (g) represent the overlap between the sampled native and nonnative climatic spaces: native climatic similarity is displayed by the overlap of the native climatic space with the nonnative, and vice versa for the nonnative climatic similarity. The colouring in all panels is based on the range (light blue, native; magenta, nonnative). Boxplots (a, d, g) show the interquartile range with the horizontal line indicating the median, and whiskers extending to the min and max values without outliers. The dashed lines represent regression lines (b, c, e, f, h, i), indicating nonsignificant relationships. The confidence intervals of the lines are presented as shadings in grey. Results are derived from linear mixed‐effect models with the variable paper set as a random effect. Details on the calculation of sampling quality parameters are provided in Fig. 2 and Supporting Information Notes S2. Individual values for each parameter and study are listed in Table S1.

Overall, the spatial coverage of the samples within both native and nonnative ranges (SpatCov_WR_) was low (mean DMC = 8.09%, SD = 5.42%; Table S1). The spatial coverage did not significantly differ between native and nonnative ranges (Fig. 3a) and showed no correlation with the number of study species (Fig. 3b) or publication year (Fig. 3c).

### Climatic coverage

To assess the climatic coverage of the sampling within both ranges, we constructed dynamic range boxes (DRB; Fig. 2.2), calculating the overlap between the sampled climatic spaces and the species' overall realised niches in its native and nonnative ranges. Dynamic range boxes provide a robust, nonparametric method to measure the size and overlap of n‐dimensional hypervolumes, such as niches and trait spaces (Junker *et al*., 2016). While plant–fungal interactions are influenced by multiple environmental factors, such as soil chemistry (Tedersoo *et al*., 2014), we focused on macroclimate due to the availability of large‐scale climatic datasets. Global Biodiversity Information Facility occurrences and sampling locations were linked to bioclimatic variables to define the overall realised climatic niche and the sampled climatic niche, respectively (Notes S2). DRB values range from 0% to 100%, with increasing values indicating an increasing overlap between the sampled climatic space and the realised niche.

The climatic coverage within ranges (ClimCov_WR_) was greater than the spatial coverage but remained low overall (mean DRB = 31.5%, SD = 25.1%, Table S1). The climatic coverage did not significantly differ between the native and the nonnative ranges of the respective study species (Fig. 3d) and showed no correlation with the number of study species (Fig. 3e) or publication year (Fig. 3f).

### Climatic similarity

To assess the climatic similarity of environmental gradients sampled in native and nonnative ranges, we used DRBs to calculate the overlap between the climatic spaces sampled in each study. This calculation was performed in both directions, as the degree of overlap depends on the relative size of the hypervolumes compared (Fig. 2.3; Notes S2).

On average, the climatic similarity (ClimSim_BR_) was low (mean = 29.4%, SD = 26.0%), yet, in a comparable quality range as the ClimCov_WR_ (Table S1). The climatic similarity did not differ depending on whether it was calculated from the native or nonnative range (Fig. 3g). Again, similarity showed no correlation with the number of study species (Fig. 3h) and did not improve over time (Fig. 3i). Note that the climatic similarity between ranges can be influenced by niche shifts that may occur during plant invasions, for example, when a species colonises regions outside its native climatic range (Atwater *et al*., 2018). To account for such shifts, we adjusted ClimSim_BR_ by weighing it according to the nonoverlap with the realised niches. While this correction increased ClimSim_BR_ values overall, it did not reveal systematic differences among studies (e.g. regarding range, number of study species, or publication year; see Fig. S5 for details).

### Summary of the findings related to sampling quality

Our analyses revealed generally low spatio‐environmental sampling coverage, particularly in comparison with a recent meta‐analysis by Lucas *et al*. (2024). This meta‐analysis evaluated the sampling quality in studies of rapid evolution published in the journal *Biological Invasions* and reported mean spatial and climatic coverage of 14.7% and 49.5%, respectively. While they interpreted these values as substantial sampling deficiencies, our results show even lower spatio‐environmental coverage in biogeographical studies on plant–fungal interactions. These findings highlight critical issues in the sampling strategies of current biogeographical studies on plant–fungal interactions.

The sampling quality was consistently low across both native and nonnative ranges. Surprisingly, we found no significant difference between studies focusing on a single species and those investigating multiple species. We expected single‐species studies to have better sampling quality, as they can allocate more resources to comprehensive sampling and are not constrained by multiple distributions. Similarly, we anticipated an improvement in sampling quality over the past 25 years, but no such trend was observed. This suggests that awareness of the need for rigorous sampling efforts remains limited when designing biogeographical studies on plant–fungal interactions.

## Implications for future biogeographical studies on plant–fungal interactions

Our systematic review identified four key limitations in biogeographical studies on plant–fungal interactions. (1) Field experiments remain scarce, despite their importance in understanding *in situ* interactions of plants and fungi (reviewed by Lekberg & Helgason, 2018). In addition to their time‐demanding nature, field experiments working with invasive species outside their native range might face legal and ethical constraints. While the deliberate introduction of invasive species to a site would be the most rigorous way to test their impact, if not feasible, field experiments can be conducted where the species already occurs. Prominent examples to implement such *in situ* experiments are the use of fungicide treatments or ingrowth cores where fungal abundance and connections to networks are manipulated (Lekberg & Helgason, 2018). (2) Many greenhouse studies relied on soil from a single location. While this approach can be appropriate for studies focusing on plant traits in response to specific fungal guilds (e.g. Seifert *et al*., 2009), it is arguably less suitable in studies where potential range shifts in soil fungal communities are of interest (e.g. Sheng *et al*., 2022). Collecting soil from multiple locations poses logistical challenges and exponentially increases sample sizes in full factorial designs. There is an obvious trade‐off between feasibility and precision, including the compromise of using pooled soil samples. However, pooling has been criticised for introducing systematic biases in direction, magnitude, and variance of plant–soil feedback studies (e.g. Reinhart & Rinella, 2016; but see Cahill *et al*., 2017; Allen *et al*., 2021). A compromise between pooling soil and a full factorial experiment where all seed sources are crossed with all soil sources is using all seed sources but exposing the plant offspring only to their ‘home’ soil and one randomly chosen soil source from the opposite range (see, e.g. Villasor *et al*., 2024). Either way, while not the main focus of our viewpoint, we emphasise that soil sampling strategies should be carefully aligned with the study's specific research questions. Additionally, we recommend assessing fungal communities in both root and soil samples across ranges to better understand biogeographical differences in the rhizosphere community and determine which parts are able to colonise the invader's roots (Řezáčová *et al*., 2022). (3) All reviewed studies focused on plants as target species. Our limited knowledge of fungal species distributions hinders the development of targeted sampling strategies for specific soil fungi taxa (e.g. Tedersoo *et al*., 2014; Mikryukov *et al*., 2023). Shifting the focus to the distribution and discreteness of individual fungal taxa could, however, significantly enhance our understanding of how biogeographical patterns in fungal distribution shape plant–fungal interactions. Large‐scale, coordinated sampling networks could help synthesise biogeographical patterns in fungal distribution. Such data could be highly valuable for future research assessing the likelihood of release from specialist soil pathogens or encounters with different and potentially more beneficial mutualists. Related to this, plant–soil feedback studies rarely characterised fungal communities to assess if differences in plant biomass correlated with particular fungal taxa and their putative function. This, and separate characterisation of fungal communities in roots and soil wherever possible, could help us understand underlying mechanisms such as defence and pathogen spillover. (4) Most studies analysed a limited number of plant populations and did not incorporate abiotic variables in their models. This data gap is central to our viewpoint, as environmental context is critical for understanding plant–fungal interactions.

As a main conclusion, we emphasise the importance of considering broad spatial and environmental sampling in future research. The critical deficiencies in the spatio‐environmental sampling quality may constrain the robustness and representativeness of native vs nonnative range comparisons (Lucas *et al*., 2024). Additionally, the low similarity between the sampled climatic gradients in the native and nonnative ranges can result in false‐positive differences between the ranges (Rosche *et al*., 2019). The extent to which the observed sampling deficiencies affected study outcomes remains uncertain, but this could be addressed through a meta‐analysis, for instance, by examining how the effect size of native vs nonnative comparisons depends on sampling quality (Colautti & Lau, 2015). We hypothesise that studies with more comprehensive sampling designs are less prone to false‐positive results.

As a hands‐on suggestion, we recommend informing sampling approaches by using approaches similar to those outlined here. The DMC provides an effective means to evaluate spatial coverage, especially when dealing with noncontiguous distributions (Sporbert *et al*., 2019). It can help identify underrepresented regions that warrant additional sampling. Similarly, the DRB can help to ensure that climatic gradients sampled in native and nonnative ranges are representative and comparable. While we used DRB to assess the realised niche of the target plant species, additional site‐specific environmental variables, particularly soil properties, should be considered. Beyond studies on plant–fungal interactions, these sampling quality parameters can be valuable tools for any biogeographical comparison, aiding in the assessment of spatial and environmental coverage.

**Fig. 4:**
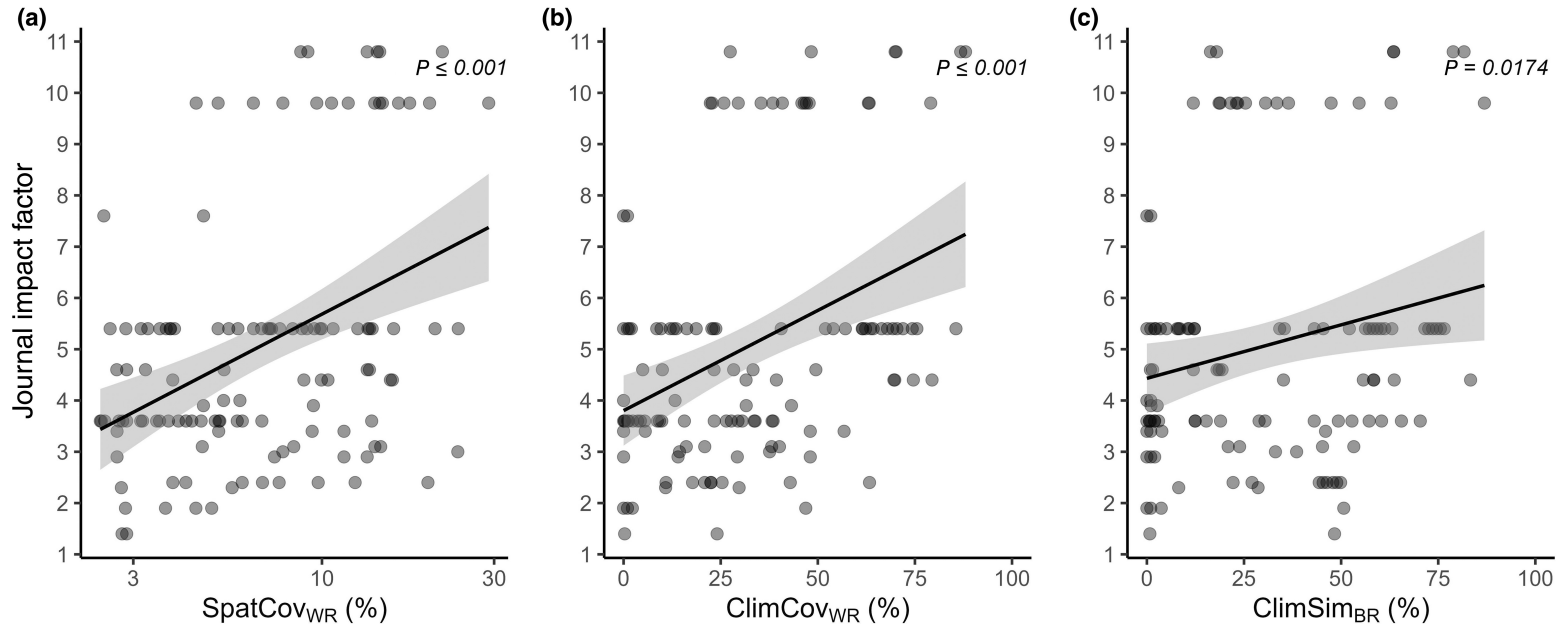
Correlation between the sampling quality and the impact factor of journals that published the reviewed studies. Correlations with impact factors are presented for (a) spatial coverage within ranges (SpatCovWR), (b) climatic coverage within ranges (ClimCovWR), and (c) climatic similarity between ranges (ClimSimBR). The current impact factor of the respective journals was retrieved from the Journal Citation Reports (clarivate.com). The solid lines represent regression lines, indicating significant relationships. The confidence intervals of the lines are presented as shadings in grey. Results are derived from linear models. Details on the calculation of sampling quality parameters are provided in Fig. 2 and Supporting Information Notes S2. Individual study values for each parameter are listed in Table S1.

Improving sampling quality increases the reliability and representativeness of results, which is widely accepted in ecology and evolution (e.g. Schweiger *et al*., 2016). Notably, we found highly significant correlations between sampling quality and journal impact factor, with studies employing more comprehensive sampling strategies being published in a higher impact journals (Fig. 4). This relationship may provide an additional incentive for researchers to refine their sampling efforts. While we acknowledge that sampling in biogeographical comparative studies presents logistical challenges, a substantial improvement is necessary. Our viewpoint is not meant as a mere criticism of the groundbreaking work done to date. Instead, by addressing current limitations and providing tools to refine sampling approaches, our viewpoint aims to improve the data quality, and consequently, the confidence in findings from native vs nonnative range comparisons.

## Competing interests

None declared.

## Author contributions

AET, YL and CR designed and planned the research. AET, DUN and MS conducted the literature review. AET and CR analysed the data. AET, YL, EW and CR wrote the manuscript with input from all co‐authors.

## Disclaimer

The New Phytologist Foundation remains neutral with regard to jurisdictional claims in maps and in any institutional affiliations.

## Supporting information


**Fig. S1** Schematic overview of the selection criteria for articles on biogeographical studies on plant–fungal interactions.
**Fig. S2** Flow diagram with detailed information on the systematic literature review process.
**Fig. S3** Density plot showing the number of plant populations sampled per range across the reviewed studies.
**Fig. S4** Schematic illustration of the scaling approach used to calculate dynamic match coefficients.
**Fig. S5** Properties of the adjusted climatic similarity (ClimSim_BR‐Adjusted_).
**Notes S1** Details on the literature review and selection of articles.
**Notes S2** Details on the quantitative analysis of the selected studies.

**Table S1** Information extracted from articles in our literature survey.
**Table S2** Targeted plant species in the 79 reviewed studies.
**Table S3** GBIF occurrences used for comparisons between overall species distribution and population sampling in the reviewed studies.
Please note: Wiley is not responsible for the content or functionality of any Supporting Information supplied by the authors. Any queries (other than missing material) should be directed to the *New Phytologist* Central Office.

## Data Availability

The data that support the findings of this study are available in the Supporting Information of this article (see Table S1).
