# Supplementary material for: Progress and future directions of biogeographical comparisons of plant–fungal interactions in invasion contexts
Source: New Phytol. 2025 May 21;247(2):477–86. doi: 10.1111/nph.70228 (PMC12177278; doi:10.1111/nph.70228)
Supplement: Supplementary file 1 — Fig. S1 Schematic overview of the selection criteria for articles on biogeographical studies on plant–fungal interactions. Fig. S2 Flow diagram with detailed information on the systematic literature review process. Fig. S3 Density plot showing the number of plant populations sampled per range across the reviewed studies. Fig. S4 Schematic illustration of the scaling approach used to calculate dynamic match coefficients. Fig. S5 Properties of the adjusted climatic similarity (ClimSimBR‐Adjusted). Notes S1 Details on the literature review and selection of articles. Notes S2 Details on the quantitative analysis of the selected studies. [file NPH-247-477-s001.pdf]

New Phytologist

***New Phytologist* Supporting Information**

Article title: Progress and future directions of biogeographical comparisons of plant-fungal interactions in invasion contexts

Authors: Arpad E. Thoma, Ylva Lekberg, Dávid U. Nagy, Min Sheng, Erik Welk, Christoph Rosche

Article acceptance date: 21 April 2025

The following Supporting Information is available for this article:

Notes S1. Details on the literature review and selection of articles.

Notes S2. Details on the quantitative analysis of the selected studies.

Figure S1. Schematic overview of the selection criteria for articles on biogeographical studies on plant-fungal interactions.

Figure S2. Flow diagram with detailed information on the systematic literature review process.

Figure S3. Density plot showing the number of plant populations sampled per range across the reviewed studies.

Figure S4. Schematic illustration of the scaling approach used to calculate Dynamic Match Coefficients (DMC).

Figure S5. Properties of the adjusted climatic similarity ($ClimSim_{BR\text{-}Adjusted}$).

Table S1. (separate .xlsx file). Information extracted from articles in our literature survey.

Table S2. Targeted plant species in the 79 reviewed studies.

Table S3. GBIF occurrences used for comparisons between overall species distribution and population sampling in the reviewed studies.

Notes S1. Details on the literature review and selection of articles

The systematic literature review was based on a Web of Science search on August 21, 2024, using the option ‘Web of Science Core Collection’ and the search query: ((fung* OR mycorrhiza* OR "arbuscular mycorrhizal fungi" OR AMF OR soil fung* OR mycobiota OR "soil biota" OR pathogen* OR "fung* disease" OR "fung* endophyt*") AND (communit* OR compositi* OR divers* OR richness OR structure OR diversity OR assemblage) AND (biogeograph* OR range OR distribution OR habitat OR location OR occurrence* OR environment) AND ((plant* OR weed* OR grass* OR legume* OR flora* OR vegetation OR wood* OR tree* OR shrub* OR herb*) NOT(animal OR invertebrate* OR vertebrate* OR mammal OR bird OR fish)) AND ((invasi* OR nonnative OR "non-native" OR "non native" OR alien OR introduced OR exotic) AND (native OR endemic OR indigenous OR natural* OR home*)).

This search query returned 1625 articles on which we conducted a title and abstract screening using the Rayyan software (Ouzzani *et al.*, 2016). To do so, we pre-defined selection criteria that were in concordance with our research objectives and followed state of the art best practices (ROSES protocol, Fig. S2) for systematic literature reviews (Haddaway *et al.*, 2018; Foo *et al.*, 2021). Specifically, we targeted studies that compared fungal community composition and function associated with a single plant species across its native and non-native ranges. In addition, we included studies investigating plant performance in response to soil fungi, either through fungicide treatments or whole-soil inoculations from these ranges. Note that we included studies utilizing whole-soil inoculations, because soil fungi constitute one of the most important groups of soil-borne pathogens and mutualists, making them likely to dominate soil biota effects (Raaijmakers *et al.*, 2009; Smith & Read, 2010; but see Torres *et al.*, 2021). The title and abstract screening resulted in 172 relevant publications. For these publications we conducted a full text screening with the same criteria as for title and abstract screening. 41 articles remained after this screening. Within each of these articles, we considered studies as independent if different plant species were investigated or distinct study types employed (e.g., a field survey and a greenhouse experiment). This resulted in a total of 79 studies within the 41 articles.

Notes S2. Details on the quantitative analysis of the selected studies

We retrieved GPS coordinates from 63 studies to quantitatively assess three sampling quality parameters: (1) Dynamic Match Coefficients (DMC; Sporbert *et al.*, 2019) to evaluate spatial coverage, (2) Dynamic Range Boxes (DRB; Junker *et al.*, 2016) to estimate climatic coverage within native and non-native ranges, and (3) the climatic similarity of sampled environmental gradients between ranges.

The GPS data originated from sampling sites of seeds and/or soil collection sites where a distinct invasive species occurred or in proximity. In 54.0% of the studies, sampling locations for plant and soil samples were identical. In 25.4% of the studies, plant and soil sampling locations differed. In such cases, we used GPS coordinates from the seed sampling. Conversely, 13 studies included plant populations from one range but sampled soil across both ranges (e.g., growing certain plant population(s) in multiple soils from the native and non-native ranges of the plant species). For these cases, we used GPS coordinates from the soil sampling (Table S2).

The spatial and climatic coverage of the samplings were set in relation to the global distribution of the plant species. To estimate this distribution, we downloaded occurrence records from GBIF. We restricted the data download to "Human Observations & Occurrences" and limited records to the time frame 2000–2024 (matching the reviewed studies). We excluded spatial outliers, defined as data points outside continuous occurrence clusters to correct for potential misidentifications, incorrect georeferencing, or planted occurrences (e.g., botanical gardens).

While GBIF is a valuable resource for species occurrences, it has known biases, particularly towards well-surveyed regions such as North America and Europe, which may affect the accuracy of species distributions (Beck *et al.*, 2014; Troia & McManamay, 2016). However, GBIF remains one of the most accessible and standardised sources for species distribution data, making it particularly useful for cross-species comparisons (García-Roselló *et al.*, 2015). For researchers seeking to apply our methods to evaluate the quality of their sampling design, we recommend using multiple data sources and accounting for spatial autocorrelation in potentially overrepresented regions (Dormann *et al.*, 2007; Pacifici *et al.*, 2017).

Our GBIF occurrence records were categorised as native or non-native occurrences based on the data base of the CABI Invasive Species Compendium (ISC; Diaz-Soltero & Scott, 2014). However, the ISC classification is relatively coarse, as it relies on country-based status assessments. This approach can be problematic, particularly in environmentally heterogeneous countries (Bindewald *et al.*, 2020). Like GBIF, the ISC provides an accessible framework for classifying occurrences as native/non-native in standardised manner across multiple taxa (Lucas *et al.*, 2024). However, for single-species range definitions, we recommend a nuanced research on the historical distribution of the study species to estimate its native and non-native ranges as precisely as possible (Rosche *et al.*, 2025).

2.1 Details on the calculation of DMC values to estimate the spatial coverage within ranges

The geographical range size of a species is defined by the latitudinal and longitudinal boundaries that delimit its spatial distribution (Gaston, 1991). We used DMC to quantify the spatial overlap between the samplings and the species distribution within each range ($SpatCov_{WR}$). DMC values estimate spatial sampling gaps across distributions and are particularly useful for non-contiguous distributions (Sporbert *et al.*, 2019). The DMC calculation is based on matches between grid cells occupied by sampling locations and grid cells of the native and non-native distributions (GBIF data). These cell matches were calculated across multiple raster resolutions, ranging from fine to coarse, with an initial cell size set to 1/20th of the species' maximum North–South and East–West range extent. Over 20 iterative scaling steps, the grid cells were progressively enlarged, and the DMC recalculated at each step (Fig. S4). As such, DMC accounts for variation in range sizes between the study species, as species with smaller ranges start with finer grain sizes, while species with larger ranges start with coarser grain sizes. The final DMC values were averaged across scaling steps, with mean values close to 100% indicating a near-complete match between spatial sampling and species distribution (Sporbert *et al.*, 2019).

2.2 Details on the calculation of DRB values to estimate climatic coverage within ranges

The realised niche of a species is conceptualised as an n-dimensional hypervolume (Hutchinson, 1957), encompassing the multi-dimensional range of abiotic and biotic conditions that support natural population persistence (Blonder *et al.*, 2018). DRB offer a robust, nonparametric approach to quantify the size and overlap of such hypervolumes (Junker *et al.*, 2016). In this study, we limited our analysis to climatic conditions, comparing the sampled niche (climatic conditions at sampling locations) with the realised global niche (climatic conditions across the species' entire distribution). To quantify these two niche spaces, we extracted raster cell values for sampling localities and GBIF occurrences separately using grid layers of 19 bioclimatic variables (BIO01–BIO19) from the WorldClim database at a spatial resolution of 10 arc seconds (Fick & Hijmans, 2017). To reduce bias from intercorrelation, we followed Cai *et al.* (2021) and excluded bioclimatic variables with an absolute pairwise correlation > 0.8. This resulted in the selection of seven variables: BIO1: Annual mean temperature, BIO2: Mean diurnal range, BIO3: Isothermality, BIO12: Annual precipitation, BIO14: Precipitation of the driest month, BIO15: Precipitation seasonality and BIO19: Precipitation of the coldest month.

The DRB approach then quantified the overlap between the climatic space of sampling locations and the realised macroclimatic niche ($ClimCov_{WR}$). Overlap values were standardised to a 0–100% scale, where values near 100% indicate a near-complete match between the sampled climatic space and the realised macroclimatic niche (Junker *et al.*, 2016).

2.3 Details on the calculation of DRB values to estimate the climatic similarity across ranges

We also applied the DRB to quantify the climatic similarity between the sampled climatic spaces of the native and non-native range ($ClimSim_{BR}$). In this between-range comparison, it is important to note that the size of the hypervolumes calculated by DRBs can vary between ranges (see Fig. 2 in the main manuscript). To account for this, we calculated climatic overlap from two perspectives: (1) The overlap of the studied native climatic space with the studied non-native climatic space and (2) the overlap the studied non-native climatic space had with the studied native climatic space.

Some species undergo climatic niche shifts between their native and non-native ranges (Atwater *et al.*, 2018), which can lead to low overall climatic similarity between the native and non-native distribution (i.e., GBIF occurrence data). To account for the potential effects of climatic niche shifts, we calculated an adjusted climatic similarity value ($ClimSim_{BR\text{-}Adjusted}$), by weighing $ClimSim_{BR}$ against the overlap of the overall possible similarity between native and non-native range of the GBIF occurrences ($ClimSim_{BR\text{-}GBIF}$): $ClimSim_{BR\text{-}Adjusted} = ClimSim_{BR} / ClimSim_{BR\text{-}GBIF}$. While the $ClimSim_{BR\text{-}Adjusted}$ values were generally higher than the $ClimSim_{BR}$ values, they did not reveal systematic differences across studies. Both values were highly correlated and showed similar patterns concerning their correlations with range, number of study species, and publication year (Fig. S5). For some studies the $ClimSim_{BR\text{-}Adjusted}$ values exceeded 100%, implying that the sampled climatic similarity extended beyond the macroclimatic niche similarity between ranges, as represented by the GBIF occurrences. This suggests that some studies sampled sites not yet included in the database. We recommend that researchers who sampled areas not yet covered by the background data, submit their findings to the database used or similar repositories to enhance data completeness and improve future analyses.

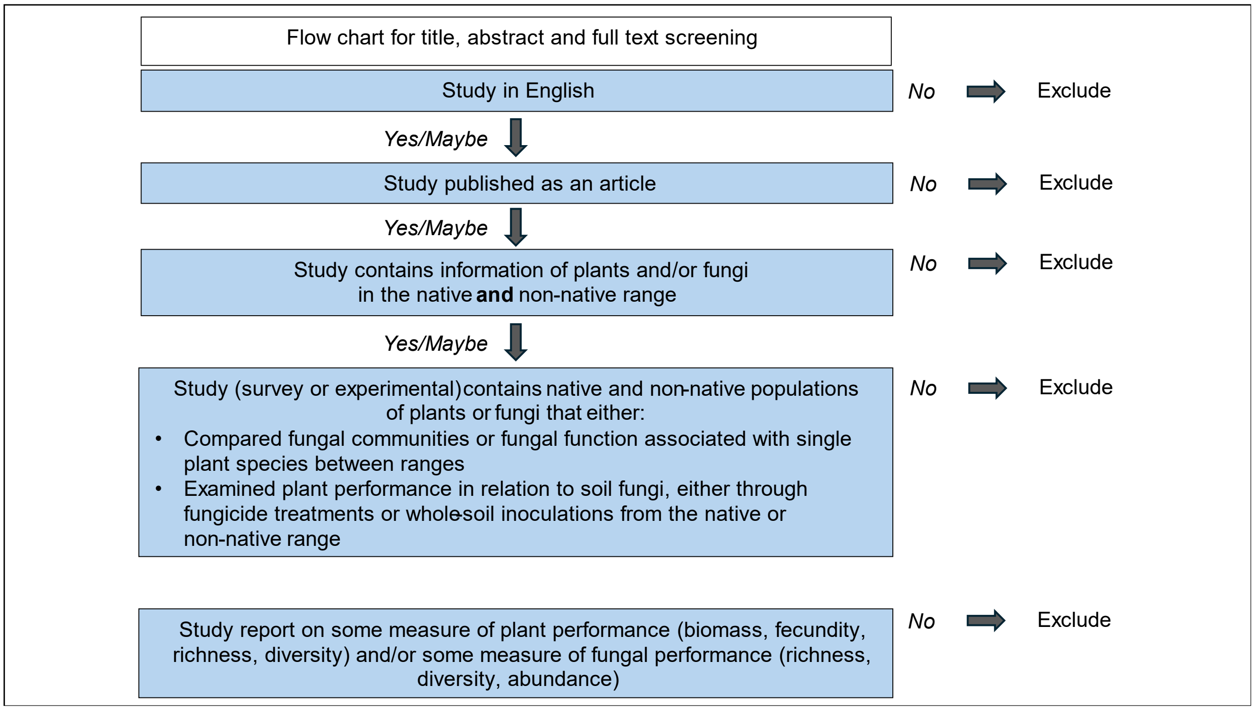

Figure S1. Schematic overview of the selection criteria for articles on biogeographical studies on plant-fungal interactions. Selection criteria were pre-defined in several discussions to meet as many articles as possible that were in concordance with our research objectives. The selection criteria were used throughout abstract and title and full-text screening.

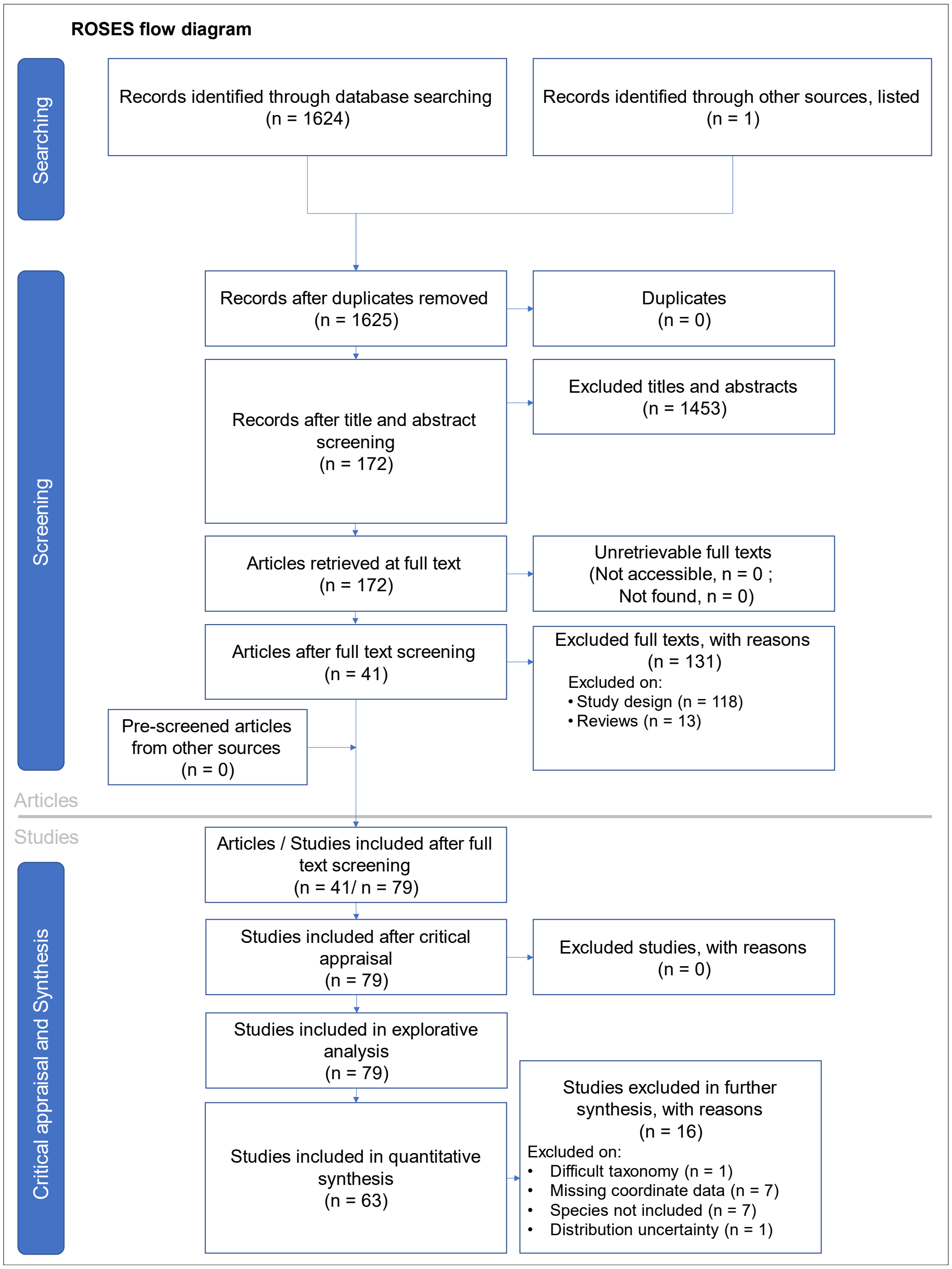

Figure S2. Flow diagram with detailed information on the systematic literature review process.

Within articles, multiple individual studies were considered if they investigated different plant species or employed different study types (e.g., a field survey and a greenhouse experiment). 79 and 63 studies were included in the explorative and quantitative analysis, respectively. For the quantitative analysis, 16 of these studies were excluded, because they did not provide sampling coordinates (n = 7), were not able to collect data

for some of the investigated plant species (n = 7), investigated a species with a diffuse global distribution (n = 1) or with uncertain taxonomy (n = 1).

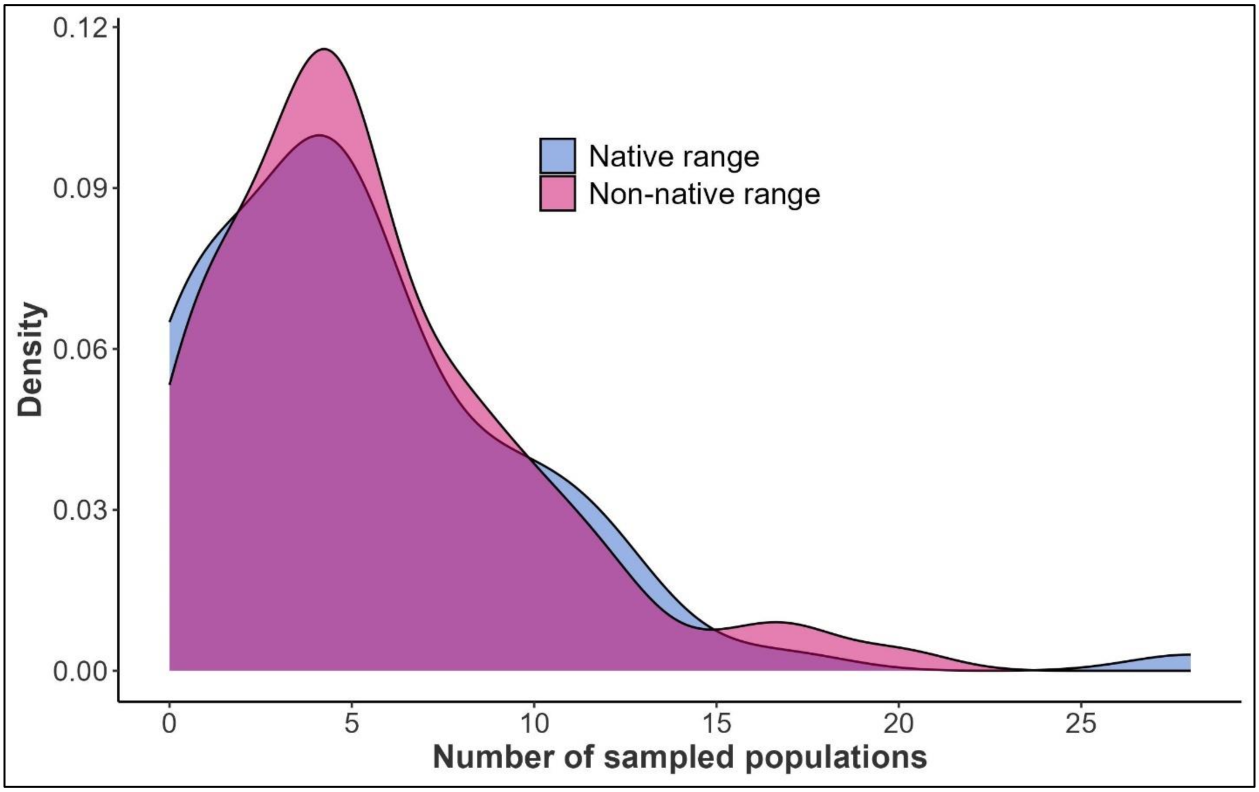

Figure S3. Density plot showing the number of plant populations sampled per range across the reviewed studies. The number of populations sampled in the native and non-native ranges is represented in light blue and magenta, respectively (see legend). The total area under each density curve equals 1 (or 100%), with peaks indicating where values are most concentrated along the x-axis (number of sampled populations per range). The plot shows that the majority of studies sampled ten or fewer populations per range, with most observations clustering around five populations per range. A complete list of studies, including the number of native and non-native populations sampled, is provided in Table S2.

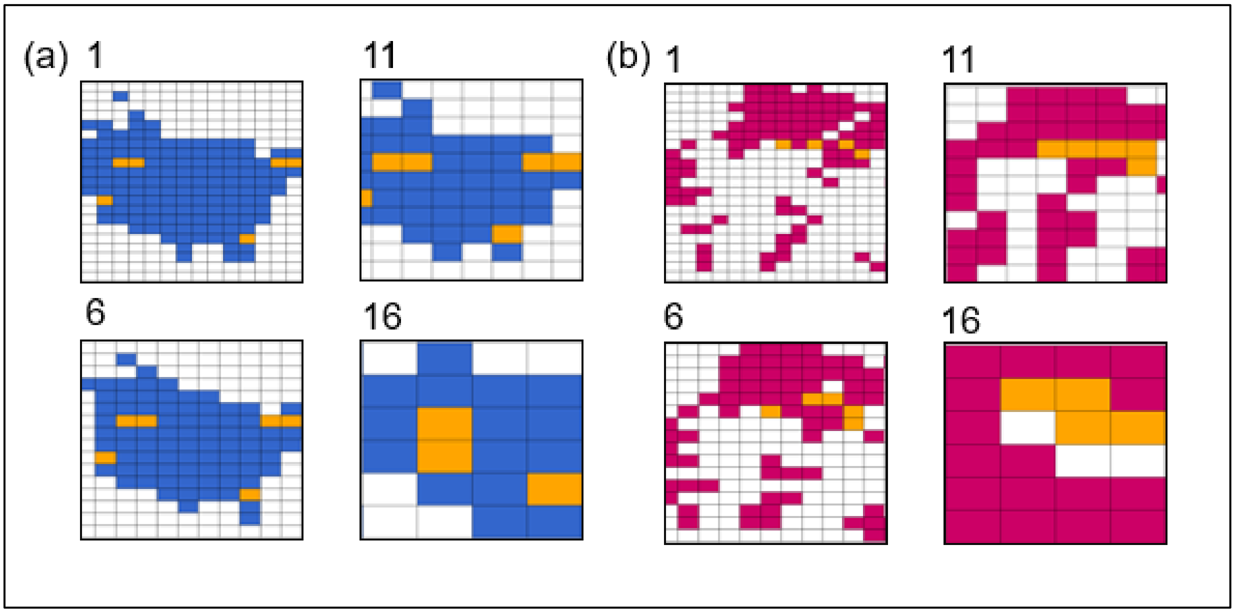

Figure S4. Schematic illustration of the scaling approach used to calculate Dynamic Match Coefficients (DMC). The example is based on a field survey by Sheng *et al.* (2022), investigating fungal associations with *Conyza canadensis* populations in the (a) native and (b) non-native ranges. The figure displays four out of 20 selected scaling steps, ranging from fine to coarse raster-cell resolution in both ranges. DMC values were calculated based on grid cell matches between the sampling locations (orange) and the native (blue) or non-native range distributions (red). Distribution data was obtained from GBIF (see Note S2 and Table S3 for details). The DMC calculation involved multiple raster resolutions, starting with an initial cell size set to 1/20th of the species' maximum North–South and East–West range extent. Over 20 iterative scaling steps, grid cells were progressively enlarged, and DMC values were recalculated at each step to derive an overall mean DMC, representing spatial sampling quality (Sporbert *et al.*, 2019).

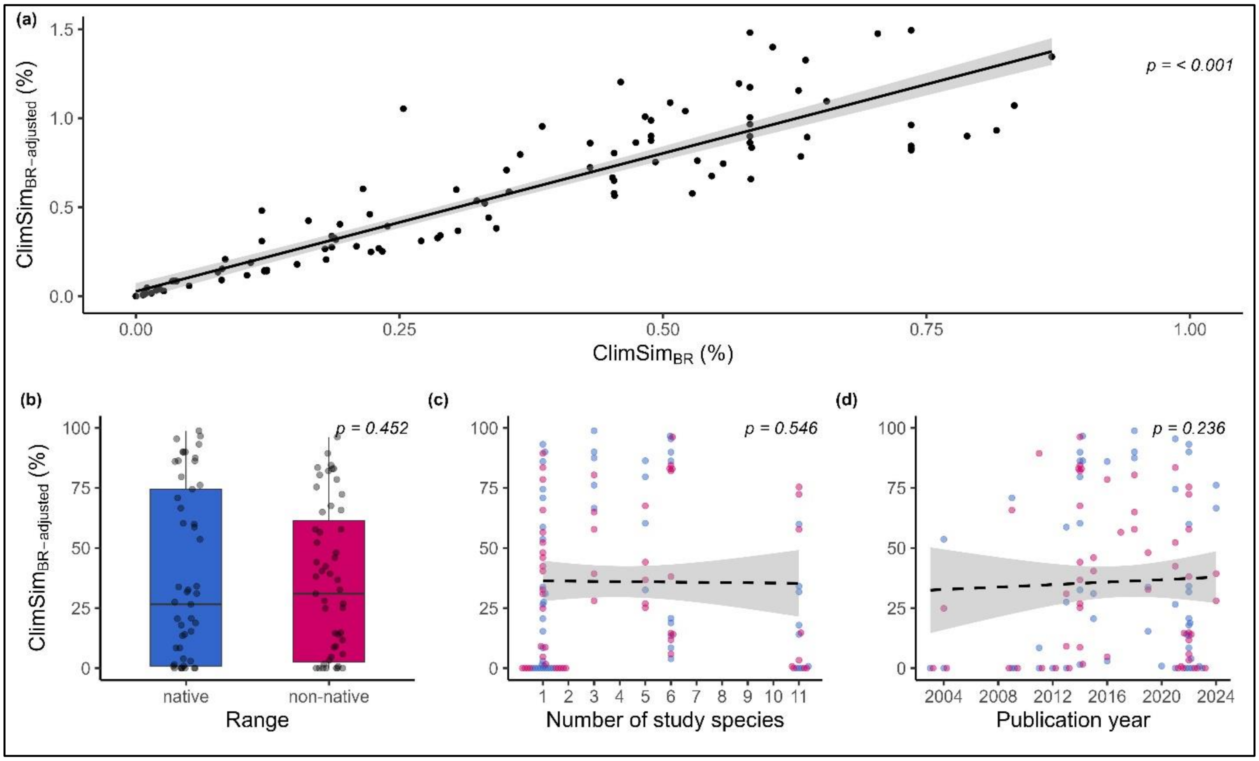

Figure S5. Properties of the adjusted climatic similarity (ClimSim$_{BR-Adjusted}$). The ClimSim$_{BR-Adjusted}$ was calculated by weighing the climatic similarity (ClimSim$_{BR}$) against the overlap of the overall possible similarity between native and non-native range of the GBIF occurrences (ClimSim$_{BR-GBIF}$): ClimSim$_{BR-Adjusted}$ = ClimSim$_{BR}$ / ClimSim$_{BR-GBIF}$. While ClimSim$_{BR-Adjusted}$ values were generally higher than ClimSim$_{BR}$ values, they did not reveal systematic differences across studies. (a) ClimSim$_{BR-Adjusted}$ and ClimSim$_{BR}$ were highly correlated to one another. Moreover, ClimSim$_{BR-Adjusted}$ showed non-significant correlations with (b) range, (c) number of study species, and (d) publication year. These relationships mirror the patterns observed for ClimSim$_{BR}$ (see in comparison Fig. 3 in the main manuscript). The colouring in panels b, c and d is based on the range (native: light blue, non-native: magenta). Boxplots (Panel b) show the interquartile range (IQR) with the horizontal line indicating the median, and whiskers extending to the min and max values without outliers. The dashed lines represent regression lines (Panels c and d), indicating non-significant relationships. The solid line in panel a represents a regression line, indicating a significant relationship. The confidence intervals of the lines are presented as shadings in grey. Results are derived from linear-mixed-effect models with the variable paper set as a random effect.

Table S2. Targeted plant species in the 79 reviewed studies. A total of 54 different plant species were investigated. Studies highlighted in grey were included in the exploratory analysis but excluded from the quantitative synthesis. Asterisks indicate 13 studies within four articles that sampled only soil samples from both ranges, while plant populations were sampled only from one range. Since these soil samples were collected at or near sites where the target species occurred, their coordinates were treated as plant species occurrence data.

Study	Targeted plant species	Native populations	Non-native populations
Andonian *et al.* (2011)	*Centaurea solstitialis*	1	3
Andonian *et al.* (2012)	*Centaurea solstitialis*	1	3
Brinbaum *et al.* (2014)	*Acacia cyclops*	5	5
Brinbaum *et al.* (2014)	*Acacia longifolia*	5	5
Brinbaum *et al.* (2014)	*Acacia melanoxylon*	5	5
Brinbaum *et al.* (2014)	*Acacia saligna*	5	5
Brinbaum *et al.* (2014)	*Paraserianthes lophantha*	5	5
Broadbent *et al.* (2018)*	*Agrostis capillaris*	5*	5*
Broadbent *et al.* (2018)*	*Anthoxanthum odoratum*	5*	5*
Broadbent *et al.* (2018)*	*Lolium perenne*	5*	5*
Callaway *et al.* (2004)*	*Centaurea stoebe*	4*	6*
Callaway *et al.* (2004)*	*Centaurea stoebe*	1*	1*
Callaway *et al.* (2011)*	*Robinia pseudoacacia*	13*	11*
Dieskau *et al.* (2019)	*Verbascum thapsus*	6	6
Dostálek *et al.* (2016)	*Rorippa austriaca*	5	5
Filep *et al.* (2021)	*Helianthus tuberosus*	28	12
Gundale *et al.* (2014)	*Pinus contorta*	0	4
Gundale *et al.* (2016)	*Pinus contorta*	2	5
Hierro *et al.* (2017)	*Centaurea solstitialis*	5	6
Johansen *et al.* (2017)	*Calamagrostis arenaria*	6	7
Knevel *et al.* (2004)	*Calamagrostis arenaria*	1	0
Lee *et al.* (2024)	*Phragmites australis*	3	3
Luo *et al.* (2021)	*Plantago virginica*	3	3
Maron *et al.* (2013)	*Centaurea stoebe*	5	5
Maron *et al.* (2014)*****	*Centaurea stoebe*	11*	10*
Maron *et al.* (2014)*****	*Euphorbia esula*	11*	10*
Maron *et al.* (2014)*****	*Hypericum perforatum*	11*	10*
Maron *et al.* (2014)*****	*Leucanthemum vulgare*	11*	10*
Maron *et al.* (2014)*****	*Linaria vulgaris*	11*	10*
Maron *et al.* (2014)*****	*Potentilla recta*	11*	10*
Maron *et al.* (2015)	*Solidago gigantea*	12	12
Moora *et al.* (2011)	*Trachycarpus fortunei*	11	3
Nakamura *et al.* (2023)	*Ardisia crenata*	3	4
Pearson *et al.* (2022)	*Hypericum perforatum*	4	5
Pearson *et al.* (2022)	*Bromus tectorum*	10	10

Pearson *et al.* (2022)	*Carduus nutans*	8	8
Pearson *et al.* (2022)	*Poa bulbosa*	10	10
Pearson *et al.* (2022)	*Potentilla recta*	7	5
Pearson *et al.* (2022)	*Rumex acetosella*	6	8
Pearson *et al.* (2022)	*Bromus tectorum*	10	10
Pearson *et al.* (2022)	*Carduus nutans*	8	8
Pearson *et al.* (2022)	*Hypericum perforatum*	4	5
Pearson *et al.* (2022)	*Poa bulbosa*	10	10
Pearson *et al.* (2022)	*Potentilla recta*	7	5
Pearson *et al.* (2022)	*Rumex acetosella*	6	8
Reinhart *et al.* (2003)	*Prunus serotina*	1	1
Reinhart *et al.* (2004)	*Acer negundo*	12	12
Reinhart *et al.* (2004)	*Acer platanoides*	8	10
Řezáčová *et al.* (2020)	*Conyza canadensis*	4	8
Rezácová *et al.* (2022)	*Ambrosia artemisiifolia*	4	3
Rezácová *et al.* (2022)	*Bothriochloa ischaemum*	4	4
Rezácová *et al.* (2022)	*Conyza canadensis*	4	7
Rezácová *et al.* (2022)	*Erigeron annus*	4	6
Rezácová *et al.* (2022)	*Lactuca serriola*	6	4
Rezácová *et al.* (2022)	*Medicago lupulina*	4	4
Rezácová *et al.* (2022)	*Plantago lanceolata*	5	4
Rezácová *et al.* (2022)	*Solidago canadensis*	4	5
Rezácová *et al.* (2022)	*Sonchus oleraceus*	4	4
Rezácová *et al.* (2022)	*Symphyotrichum lanceolatum*	4	4
Rezácová *et al.* (2022)	*Trifolium repens*	4	4
Seifert *et al.* (2009)	*Hypericum perforatum*	14	15
Shah *et al.* (2015)*	*Conyza canadensis*	3*	3*
Sheng *et al.* (2022)	*Conyza canadensis*	17	17
Sheng *et al.* (2022)	*Conyza canadensis*	12	17
Sun *et al.* (2014)	*Centaurea stoebe*	3	4
Tanner *et al.* (2014)	*Impatiens glandulifera*	2	3
te Beest *et al.* (2009)	*Chromolaena odorata*	1	1
te Beest *et al.* (2009)	*Chromolaena odorata*	3	3
Tian *et al.* (2021)	*Triadica sebifera*	12	10
Uddin *et al.* (2021)	*Lonicera japonica*	9	10
Van Grunsven *et al.* (2009)	*Carpobrotus edulis*	1	2
Villasor *et al.* (2024)	*Agropyron cristatum*	7	7
Villasor *et al.* (2024)	*Bromus inermis*	8	8
Villasor *et al.* (2024)	*Poa angustifolia*	9	8
Waller *et al.* (2016)	*Centaurea solstitialis*	12	12
Yang *et al.* (2013)	*Triadica sebifera*	8	8
Yang *et al.* (2019)	*Triadica sebifera*	6	6
Yang *et al.* (2022)	*Triadica sebifera*	3	3
Zhang *et al.* (2013)	*Triadica sebifera*	4	4

Table S3. GBIF occurrences used for comparisons between overall species distribution and population sampling in the reviewed studies. Data sets are available for download through the provided links.

Species	Reference
Acacia cyclops	GBIF.org (03 September 2024): https://doi.org/10.15468/dl.zanwqa
Acacia saligna	GBIF.org (03 September 2024) : https://doi.org/10.15468/dl.xur5v9
Acacia longifolia	GBIF.org (3 September 2024) : https://doi.org/10.15468/dl.ybhjpp
Acacia melanoxylon	GBIF.org (3 September 2024) : https://doi.org/10.15468/dl.n2wnvs
Paraserianthes lophantha	GBIF.org (03 September 2024) : https://doi.org/10.15468/dl.3xht8c
Centaurea stoebe	GBIF.org (03 September 2024) : https://doi.org/10.15468/dl.za2una
Verbascum thapsus	GBIF.org (03 September 2024) : https://doi.org/10.15468/dl.zwkbu6
Helianthus tuberosus	GBIF.org (03 September 2024) : https://doi.org/10.15468/dl.37nszv
Calamagrostis arenaria	GBIF.org (17 September 2024) : https://doi.org/10.15468/dl.j5ugtu
Conyza canadensis	GBIF.org (05 September 2024) : https://doi.org/10.15468/dl.y9jegy
Hypericum perforatum	GBIF.org (03 September 2024) : https://doi.org/10.15468/dl.fmuqy2
Impatiens glandulifera	GBIF.org (03 September 2024) : https://doi.org/10.15468/dl.dwssmx
Centaurea solstitialis	GBIF.org (17 September 2024) : https://doi.org/10.15468/dl.v6uw94
Triadica sebifera	GBIF.org (04 September 2024) : https://doi.org/10.15468/dl.r45rev
Ardisia crenata	GBIF.org (04 September 2024) : https://doi.org/10.15468/dl.vyvb2g
Trachycarpus fortunei	GBIF.org (04 September 2024) : https://doi.org/10.15468/dl.nknatg
Bromus tectorum	GBIF.org (17 September 2024) : https://doi.org/10.15468/dl.4cuvp8
Poa bulbosa	GBIF.org (17 September 2024) : https://doi.org/10.15468/dl.gfntg6
Carduus nutans	GBIF.org (17 September 2024) : https://doi.org/10.15468/dl.xs4xr6
Solidago gigantea	GBIF.org (09 November 2024) : https://doi.org/10.15468/dl.ztbumn
Rorippa austriaca	GBIF.org (09 November 2024) : https://doi.org/10.15468/dl.4xpgre
Chromolaena odorata	GBIF.org (09 November 2024) : https://doi.org/10.15468/dl.2nr6pn
Lonicera japonica	GBIF.org (09 November 2024) : https://doi.org/10.15468/dl.2gmpb9
Plantago virginica	GBIF.org (09 November 2024) : https://doi.org/10.15468/dl.89hj8r
Solidago canadensis	GBIF.org (09 November 2024) : https://doi.org/10.15468/dl.8krrzt
Erigeron annuus	GBIF.org (09 November 2024) : https://doi.org/10.15468/dl.xjy4a3
Medicago lupulina	GBIF.org (09 November 2024) : https://doi.org/10.15468/dl.p7ak4e
Symphyotrichum lanceolatum	GBIF.org (09 November 2024) : https://doi.org/10.15468/dl.xgvmbf
Plantago lanceolata	GBIF.org (09 November 2024) : https://doi.org/10.15468/dl.hekkkj
Ambrosia artemisiifolia	GBIF.org (09 November 2024) : https://doi.org/10.15468/dl.ybseue
Lactuca seriola	GBIF.org (09 November 2024) : https://doi.org/10.15468/dl.sjuyjg
Bothriochloa ischaemum	GBIF.org (09 November 2024) : https://doi.org/10.15468/dl.rcs73u

Trifolium repens	GBIF.org (09 November 2024) : https://doi.org/10.15468/dl.fyzw8j
Sonchus oleraceus	GBIF.org (09 November 2024) : https://doi.org/10.15468/dl.3g8uur
Agropyron cristatum	GBIF.org (09 November 2024) : https://doi.org/10.15468/dl.eazhgq
Bromus inermis	GBIF.org (10 November 2024) : https://doi.org/10.15468/dl.d7n2u7
Prunus serotina	GBIF.org (25 November 2024) : https://doi.org/10.15468/dl.d6rc6p
Robinia pseudoacacia	GBIF.org (25 November 2024) : https://doi.org/10.15468/dl.7useyx
Euphorbia esula	GBIF.org (25 November 2024) : https://doi.org/10.15468/dl.cep3ew
Linaria vulgaris	GBIF.org (25 November 2024) : https://doi.org/10.15468/dl.zxas3k
Leucanthemum vulgare	GBIF.org (25 November 2024) : https://doi.org/10.15468/dl.x6t54s
Agrostis capillaris	GBIF.org (25 November 2024) : https://doi.org/10.15468/dl.fereng
Lolium perenne	GBIF.org (25 November 2024) : https://doi.org/10.15468/dl.9zeqsh
Anthoxanthum odoratum	GBIF.org (25 November 2024) : https://doi.org/10.15468/dl.7tejug

References

Atwater DZ, Ervine C, Barney JN. 2018. Climatic niche shifts are common in introduced plants. *Nature Ecology & Evolution* **2**: 34–43.

Beck J, Böller M, Erhardt A, Schwanghart W. 2014. Spatial bias in the GBIF database and its effect on modeling species' geographic distributions. *Ecological Informatics* **19**: 10–15.

Bindewald A, Michiels H-G, Bauhus J. 2020. Risk is in the eye of the assessor: comparing risk assessments of four non-native tree species in Germany. *Forestry: An International Journal of Forest Research* **93**: 519–534.

Blonder B, Morrow CB, Maitner B, Harris DJ, Lamanna C, Violle C, Enquist BJ, Kerkhoff AJ. 2018. New approaches for delineating n-dimensional hypervolumes. *Methods in Ecology and Evolution* **9**: 305–319.

Cai Q, Welk E, Ji C, Fang W, Sabatini FM, Zhu J, Zhu J, Tang Z, Attorre F, Campos JA, *et al.* 2021. The relationship between niche breadth and range size of beech (Fagus) species worldwide. *Journal of Biogeography* **48**: 1240–1253.

Diaz-Soltero H, Scott PR. 2014. Global identification of invasive species: the CABI Invasive Species Compendium as a resource. In: CABI Invasives Series. Invasive species and global climate change. 232–239.

Dormann C, M. McPherson J, B. Araújo M, Bivand R, Bolliger J, Carl G, G. Davies R, Hirzel A, Jetz W, Daniel Kissling W, *et al.* 2007. Methods to account for spatial autocorrelation in the analysis of species distributional data: a review. *Ecography* **30**: 609–628.

Fick SE, Hijmans RJ. 2017. WorldClim 2: new 1-km spatial resolution climate surfaces for global land areas. *International Journal of Climatology* **37**: 4302–4315.

Foo YZ, O'Dea RE, Koricheva J, Nakagawa S, Lagisz M. 2021. A practical guide to question formation, systematic searching and study screening for literature reviews in ecology and evolution. *Methods in Ecology and Evolution* **12**: 1705–1720.

García-Roselló E, Guisande C, Manjarrés-Hernández A, González-Dacosta J, Heine J, Pelayo-Villamil P, González-Vilas L, Vari RP, Vaamonde A, Granado-Lorencio C, *et al.* 2015. Can we derive macroecological patterns from primary Global Biodiversity Information Facility data? *Global Ecology and Biogeography* **24**: 335–347.

Gaston KJ. 1991. How Large Is a Species' Geographic Range? *Oikos* **61**: 434–438.

Haddaway NR, Macura B, Whaley P, Pullin AS. 2018. ROSES RepOrting standards for Systematic Evidence Syntheses: pro forma, flow-diagram and descriptive summary of the plan and conduct of environmental systematic reviews and systematic maps. *Environmental Evidence* **7**: 7.

Hutchinson GE. 1957. Concluding Remarks. *Cold Spring Harbor Symposia on Quantitative Biology* **22**: 415–427.

Junker RR, Kuppler J, Bathke AC, Schreyer ML, Trutschnig W. 2016. Dynamic range boxes – a robust nonparametric approach to quantify size and overlap of n-dimensional hypervolumes. *Methods in Ecology and Evolution* **7**: 1503–1513.

Lucas MS, Hensen I, Barratt CD, Callaway RM, Durka W, Lekberg Y, Nagy DU, Onstein RE, Shah MA, van Dam NM, *et al.* 2024. Re-focusing sampling, design and experimental methods to assess rapid evolution by non-native plant species. *Biological Invasions* **26**: 1327–1343.

Ouzzani M, Hammady H, Fedorowicz Z, Elmagarmid A. 2016. Rayyan—a web and mobile app for systematic reviews. *Systematic Reviews* **5**: 210.

Pacifici K, Reich BJ, Miller DAW, Gardner B, Stauffer G, Singh S, McKerrow A, Collazo JA. 2017. Integrating multiple data sources in species distribution modeling: a framework for data fusion. *Ecology* **98**: 840–850.

Raaijmakers JM, Paulitz TC, Steinberg C, Alabouvette C, Moënne-Loccoz Y. 2009. The rhizosphere: a playground and battlefield for soilborne pathogens and beneficial microorganisms. *Plant and Soil* **321**: 341–361.

Rosche C, Broennimann O, Novikov A, Mrázová V, Boiko GV, Danihelka J, Gastner MT, Guisan A, Kožić K, Lehnert M, *et al.* 2025. Herbarium specimens reveal a cryptic invasion of polyploid Centaurea stoebe in Europe. *New Phytologist* **245**: 392–405.

Smith SE, Read DJ. 2010. *Mycorrhizal Symbiosis*. Cambridge, UK: Academic Press.

Sporbert M, Bruelheide H, Seidler G, Keil P, Jandt U, Austrheim G, Biurrun I, Campos JA, Čarni A, Chytrý M, *et al.* 2019. Assessing sampling coverage of species distribution in biodiversity databases. *Journal of Vegetation Science* **30**: 620–632.

Torres N, Herrera I, Fajardo L, Bustamante RO. 2021. Meta-analysis of the impact of plant invasions on soil microbial communities. *BMC Ecology and Evolution* **21**: 172.

Troia MJ, McManamay RA. 2016. Filling in the GAPS: evaluating completeness and coverage of open-access biodiversity databases in the United States. *Ecology and Evolution* **6**: 4654.
